# Fabricating Superior NiAl Bronze Components through Wire Arc Additive Manufacturing

**DOI:** 10.3390/ma9080652

**Published:** 2016-08-03

**Authors:** Donghong Ding, Zengxi Pan, Stephen van Duin, Huijun Li, Chen Shen

**Affiliations:** School of Mechanical, Materials, and Mechatronics Engineering, Faculty of Engineering and Information Science, University of Wollongong, Northfield Ave, Keiraville, NSW 2500, Australia; donghong@uow.edu.au (D.D.); svanduin@uow.edu.au (S.v.D.); huijun@uow.edu.au (H.L.); cs395@uowmail.edu.au (C.S.)

**Keywords:** NiAl bronze, additive manufacturing, arc welding, mechanical properties, microstructures

## Abstract

Cast nickel aluminum bronze (NAB) alloy is widely used for large engineering components in marine applications due to its excellent mechanical properties and corrosion resistance. Casting porosity, as well as coarse microstructure, however, are accompanied by a decrease in mechanical properties of cast NAB components. Although heat treatment, friction stir processing, and fusion welding were implemented to eliminate porosity, improve mechanical properties, and refine the microstructure of as-cast metal, their applications are limited to either surface modification or component repair. Instead of traditional casting techniques, this study focuses on developing NAB components using recently expanded wire arc additive manufacturing (WAAM). Consumable welding wire is melted and deposited layer-by-layer on substrates producing near-net shaped NAB components. Additively-manufactured NAB components without post-processing are fully dense, and exhibit fine microstructure, as well as comparable mechanical properties, to as-cast NAB alloy. The effects of heat input from the welding process and post-weld-heat-treatment (PWHT) are shown to give uniform NAB alloys with superior mechanical properties revealing potential marine applications of the WAAM technique in NAB production.

## 1. Introduction

Cast nickel aluminum bronze (NAB) alloys are widely used for marine applications due to their excellent mechanical properties and corrosion resistance. It also has good abrasion resistance, which makes NAB alloy an ideal material for landing gear bushings and bearings. As summarized by Brezina [1], NAB is a copper-based quaternary alloy that typically contains 8–12 wt % aluminum, 3–6 wt % nickel and iron. It has a two-phase microstructure, an equilibrium fcc α phase and a high-temperature bcc β phase. NAB has enhanced mechanical properties due to the addition of nickel and iron to copper-aluminum alloys, which increase its mechanical properties through the precipitation of several intermetallics κ among the α and the β phases. In addition, the low content of manganese (max 3.5 wt %) in commercial NAB alloys improves its casting ability and acts as a microstructure stabilizer. Apart from mechanical properties, NAB alloys are characterized by their excellent corrosion behavior, partially because Ni and Fe extend the terminal α phase field and suppress the γ_2_ phase formation that occurs in binary Cu-Al alloys. Culpan and Rose [2] claimed that the absence of the γ_2_ phase is preferable as it is a hard and brittle phase containing high Al content that impairs the material’s corrosion resistance. Additionally, Yu et al. [3] found the formation of a tough aluminum oxide film (Al_2_O_3_) gives NAB its excellent corrosion and erosion cavitation resistance. 

In marine applications however, Ferrara and Caton [4] summarized that cast components usually have thick sections, and the resulting slow cooling rates (typically 10^−3^ to 10^−2^ °C/s) contribute to coarse microstructures and reduced mechanical properties. This is coupled with the crucial problem of casting porosity which further reduces mechanical properties and service performance of cast NAB [5].

Several methods for improving the microstructure and mechanical properties of NAB e.g., heat treatment, friction stir processing (FSP), and fusion welding, are reported in the literature. The microstructures and corresponding hardness of NAB material are strongly dependent on the applied heat treatment. Wu et al. [6] investigated that different peak temperatures and cooling rates form various phases (i.e., Widmanstätten α, bainitic α, or even martensitic β) resulting in different mechanical properties, as well as corrosion behavior. However, in marine application, some large cast components with varied thicknesses may not be heat-treatable. FSP was extensively performed to selectively modify the near-surface layers of cast NAB components. Fuller et al. [5] achieved microstructure refinement and homogenization, as well as closure of porosity of as-cast NAB materials through FSP. Nevertheless, this modification method is only available for near-surface layers rather than for through-thickness components due to the shallow pin depth of the FSP tools [7]. Fusion welding was often implemented to repair damaged NAB components. Li et al. [8] investigated the effect of welding parameters, such as current, voltage, wire-feed rate and travel speed, on the formation of the weld bead. It is found that due to the rapid cooling rate (around 1 °C/s), α with a Widmanstätten morphology, as well as bainitic and martensitic products of β phase may become apparent, which exhibit both higher strength and ductility of the material than the slowly cooled cast metal.

Recently, wire arc additive manufacturing (WAAM) techniques, using either the gas metal arc welding (GMAW) or gas tungsten arc welding (GTAW) processes, have been developed for layer-by-layer manufacturing of functional metal components with full density [9,10,11]. Ding et al. [12] reviewed the technologies and developments of wire-feed additive manufacturing of metal components and revealed that WAAM becomes a promising alternative for fabricating components with medium to large size due to its productivity, cost-competitiveness, energy efficiency, and unlimited build envelop. For aerospace applications, wall structures of titanium alloys have been built using WAAM, and their mechanical properties were demonstrated to be comparable to cast or wrought materials [13]. In comparison to casting technology, firstly the additive manufacturing (AM) technique offers a great potential for time and cost savings, especially for newly-designed components. Secondly, it is able to fabricate complex shapes owing to its paradigm shift through layer-by-layer manufacturing. Lastly, as demonstrated by Shen et al. [14], AM techniques make it possible to build intermetallic materials and functional graded materials, which poses challenges using traditional casting technology.

At present, there has not been any work reported for manufacturing of NAB alloys using the WAAM process. Investigation of the WAAM of NAB may provide an alternative production method to significantly reduce manufacturing lead times as well as overcoming common defects trapped inside cast components. This study focuses on the WAAM of NAB components. Firstly, thin wall samples are fabricated to investigate the feasibility of the WAAM of NAB. Then, microstructure and mechanical properties of additively manufactured NAB wall structures are compared to as-cast NAB. Finally, the effects of heat inputs and post-weld-heat-treatment (PWHT), on the properties of weld metal (WM), are analyzed and discussed. Other properties, like erosion-corrosion, wear and high temperature behavior of the NAB alloy, are beyond the scope of this paper. However, corrosion behavior was intensively investigated and can be found elsewhere [15,16].

## 2. Experimental Procedure

**Figure 1:**
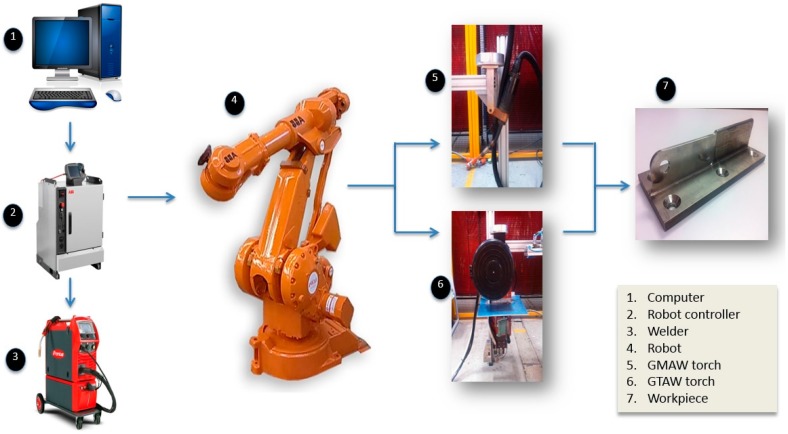
Schematic diagram of the developed experimental WAAM system.

An experimental robotic WAAM system was developed as shown in Figure 1. A computer interface (1) is used to program the experimental processes. The robot controller (2) is used to coordinate both the welding power source (3) and the robot motions (4). Either a GMAW (5) or GTAW torch (6) could be used to deliver the wire arc depending on experimental requirements. An example of an AM work-piece is shown in (7).

**Figure 2:**
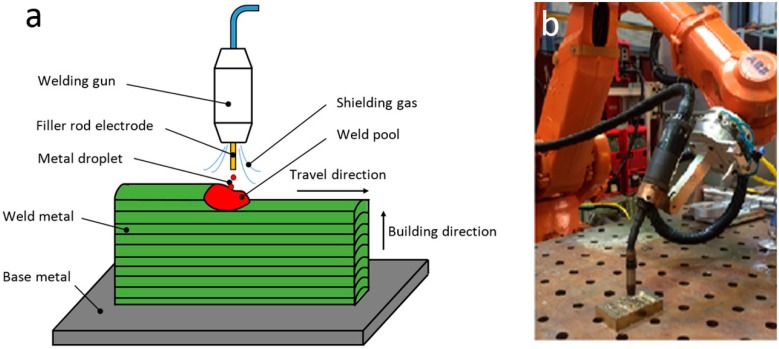
(**a**) Schematic diagram of layer-by-layer deposition using GMAW process; and (**b**) the setup of the robotic GMAW process.

In this study, GMAW process is performed due to its high deposition rate (up to 5 kg/h in the present study). A synergic pulse spray transfer mode and argon shielding gas were used for welding. Figure 2a shows the schematic diagram of layer-by-layer additive manufacturing process using GMAW. A molten metal pool is sustained when an electric arc forms between a consumable wire electrode and work-piece metal (base metal or previous-deposited weld metal). Welding wire is fed into the molten pool under the protection of the shielding gas. When moving the welding torch with controlled travel speeds along predefined orientations and paths, geometrical metal objects are fabricated layer-by-layer. Figure 2b shows the setup of the robotic GMAW process.

**Figure 3:**
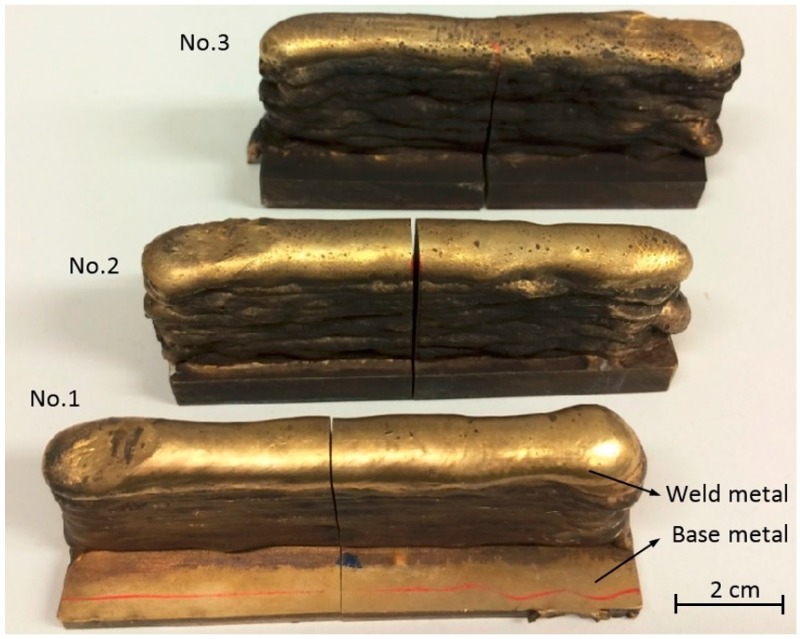
Three additively-manufactured NAB components. The deposited wall components were wire-cut from the middle in the lengthwise direction with one half of the walls further processed with post-weld-heat-treatment.

To illustrate the fabrication of NAB alloy, Figure 3 shows three testing wall structures deposited on as-cast base metals using WAAM. The composition of NAB base metal (BM) and welding wire are listed in Table 1, showing the similar composition of materials, specifically the content of aluminum, nickel, and iron. The as-cast BM was made by Veem Ltd., Perth, Australia according to the Australia Defense Standard NES 747 specifications. Commercial 1.2 mm ALUNOX AX-CuAl8Ni6 welding wire was used. The welding parameters used are listed in Table 2, in which three different wire-feed rates were applied to test the effects of heat inputs on microstructures and mechanical properties of additively manufactured NAB components. No BM preheating was conducted prior to commencing the deposition process; therefore, the cooling rate of the first deposited layer was maximum. With the increasing height of the deposited wall, the cooling rate decreases. The deposited wall components (Figure 3) typically have a length of 100 mm and a height of 40 mm. With the increasing of heat input from 653 J/mm to 1114 J/mm in Table 2, the thicknesses of the walls increased from ~10 mm to ~14 mm.

**Figure 4:**
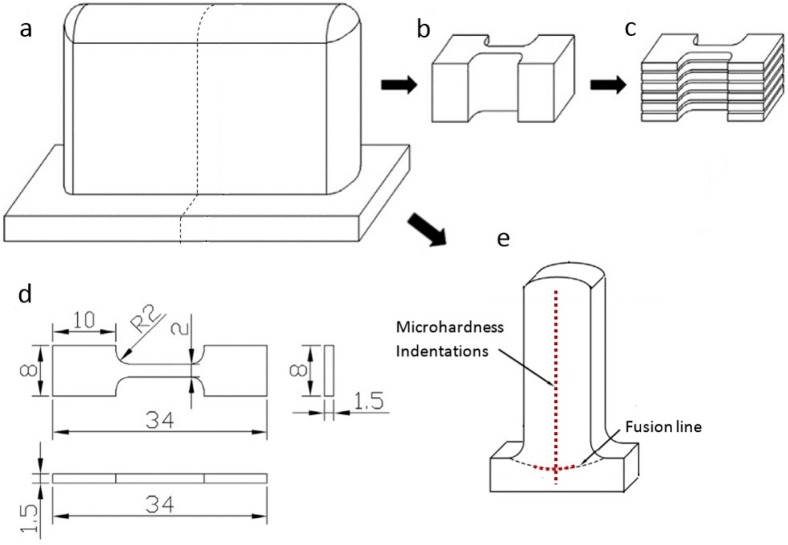
Sample preparation: (**a**) wall structure and the middle cutting trajectory; (**b**) bulk tensile sample cut from the sub-wall; (**c**) tensile specimens after slicing bulk sample; (**d**) dimensions of miniature tensile specimens in mm; and (**e**) wire-cut cross-section for microstructure observations and hardness testing.

Figure 4 shows the sample preparation procedures. Each wall was wire-cut into two sub-walls with one half post-weld-heat-treated for comparison, as shown in Figure 4a. Annealing treatment of 675 °C for six hours, followed by air cooling, was used to release the induced residual stresses from the welding process. For each sub-wall, the bulk tensile sample was cut (see Figure 4b), and then more than 10 specimens were sliced and numbered from the bottom to the top covering the region of BM and WM, as shown in Figure 4c. A total of around 80 miniature specimens cut from six sub-walls were obtained and performed tensile testing. The detailed dimensions of the specimens used are shown in Figure 4d. Cross-sections of each sub-wall were cut out with a 2 mm wire saw for microstructure observations and hardness testing. Microhardness was tested for all cross-sectioned samples along the vertical centerline and the heat-affected zones just under the fusion line, as indicated in Figure 4e. 

The metallographic specimens were prepared using standard procedures for NAB. A mixed solution of 5 vol % ferric chloride, 25 vol % hydrochloric acid, and 70 vol % distilled water was selected as the etching agent. The microstructure of the specimens were examined using scanning electron microscopy (SEM, JEOL JSM-6490LA, Tokyo, Japan). A GBC MMA X-ray diffractometer (XRD) (GBC Scientific Equipment, Chicago, IL, USA) with CuKα radiation (λ = 1.5418 Å) was used to identify the phase constitutions. Vickers microhardness profiles were measured at a load of 1 kg (HV1) by a DuraScan Hardness Tester. Tension tests were performed on a MTS370 (MTS Systems Corporation, Minneapolis, MN, USA) load unit at a strain rate of 1.8 × 10^−3^ s^−1^ at room temperature.

## 3. Results and Discussion

**Figure 5:**
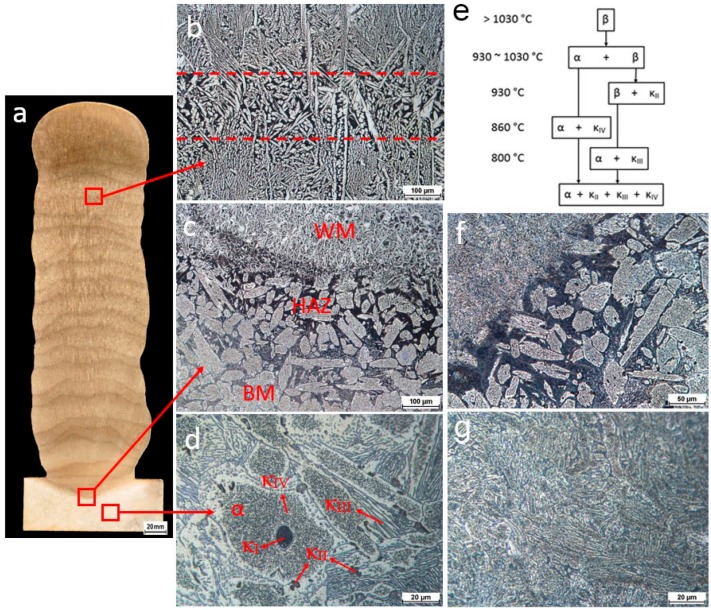
Microstructure of the deposited NAB components: (**a**) macro photography of the cross-section of the wall structure; (**b**) representative microstructure in the weld metal; (**c**) morphology of the HAZ; (**d**) representative microstructure in the base metal; (**e**) formation of transformed products in NAB alloys; (**f**) typical microstructure of the HAZ after PWHT; and (**g**) typical microstructure of the weld metal after PWHT.

Fully-dense NAB components are obtained, as the macro photography of the cross-section shows in Figure 5a. The regions of BM, heat affected zone (HAZ), and WM are found from the bottom to the top of the cross-section. In the region of WM, a series of layer bands can be readily observed. This phenomenon is well documented in a number of studies on different AM processes. Microstructures of different regions are shown in Figure 5b–d and the details are provided in the following paragraphs.

Figure 5b shows the representative microstructure of WM. The as-deposited WM contains predominantly Widmanstätten α and very fine martensite (dark areas). It was fairly fine and uniform. The layer band of reheated weld metal (area between two dashed lines in the Figure 5b) is very narrow, with the thickness of ~200 µm, and the distance between each area of reheated weld metal is ~2 mm. The number of layer bands in additively manufactured NAB is found to be exactly equal to the number of deposition passes. This means the average layer thickness is approximately 2 mm and the height of the wall is approximately 40 mm. This is consistent with the experimental measurements. The final layer thickness is approximately 3 mm indicating that the previous layer is partially re-melted by the deposition of the preceding layer. Without the further re-melting process, the thickness of the last layer is larger than the average layer thickness. Layer bands are a kind of HAZ, which results from the sub-melting-point reheating treatment that was detailed by Liu et al. [17]. 

Figure 5c shows the microstructure of the HAZ. The width of the HAZ is very narrow (<1 mm) for all welding parameters. The microstructure underwent significant changes in the HAZ. A transformation of eutectic structure to martensite occurred, in particular in the area close to the fusion line. The peak temperature in this area was above the eutectic point, resulting in the formation of a β phase. This was retained with rapid cooling and the subsequent formation of a martensitic structure. The changing temperature gradient, with distance to the fusion line, led to regions of a partially transformed eutectic structure. The martensitic structure is harder than the α phase, but are also more brittle and susceptible to corrosion and cavitation erosion, making it undesirable.

Figure 5d shows a typical microstructure of as-cast BM consisting of α phase and various κ intermetallic phases. During slow cooling (~10^−3^ °C∙s^−1^) to room temperature, the NAB alloy undergoes several microstructure changes as shown in Figure 5e. The microstructure is entirely bcc β phase at high temperature. At the temperature around 1030 °C, the primary fcc Cu-rich α phase starts to form within the β phase. When the temperature falls until 930 °C, the dendritic (variously called globular or rosette)-shaped κ_II_ phase begins to form and is normally distributed at the α/β interface. The κ_II_ precipitates are based on the Fe_3_Al (DO3 structure) and typically around 5 to 10 µm. In NABs with a high iron content of greater than ~5% and/or a high Fe to Ni ratio, the large dendritic shaped κ_I_ phase, typically 20 to 50 µm in diameter, would form and mostly locate in the centers of α grains as described by Hasan et al. [18]. A small amount of κ_I_ precipitates were found in the present cast base metal as shown in Figure 5d since the weight of iron is close to 5%. This phase is also in a form of Fe_3_Al (DO3 structure), but much coarser than the κ_II_ phase. Around the temperature below 860 °C, the much finer κ_IV_ phase begins to form in the α phase since the solubility of iron is exceeded. The cuboidal or cruciform shaped κ_IV_ precipitates are also based on the Fe_3_Al (DO3 structure). At the temperature of 800 °C, the remaining β phase is transformed into the intermetallic κ_III_ phase through the eutectoid reaction β → α + κ_III_. The κ_III_ phase, which is based on NiAl (B2 structure), typically has a lamellar morphology and formed at the α/β boundary. Once the cast NAB alloy is slowly cooled to room temperature, the alloy entirely contains the α phase and the several κ intermetallic phases.

PWHT relieves the residual stresses and homogenizes the microstructure. The BM contained a stable casting structure; therefore, little to no change occurred with PWHT. The HAZ, where martensite was present, was refined as shown in Figure 5f. The HAZ retained large sections of α phase, however, closer to the fusion line appeared partially decomposed. Additionally, the martensite was replaced with a fine grain α phase and κ precipitates. WM has a structure with Widmanstätten α and a small amount of martensite, whilst the PWHT decomposed these leaving a fine homogenized microstructure of α phase and κ precipitates as shown in Figure 5g. The refinement that occurred in both the HAZ and the WM was favorable. A fine microstructure provides additional strength to the material and improved corrosion resistance.

**Figure 6:**
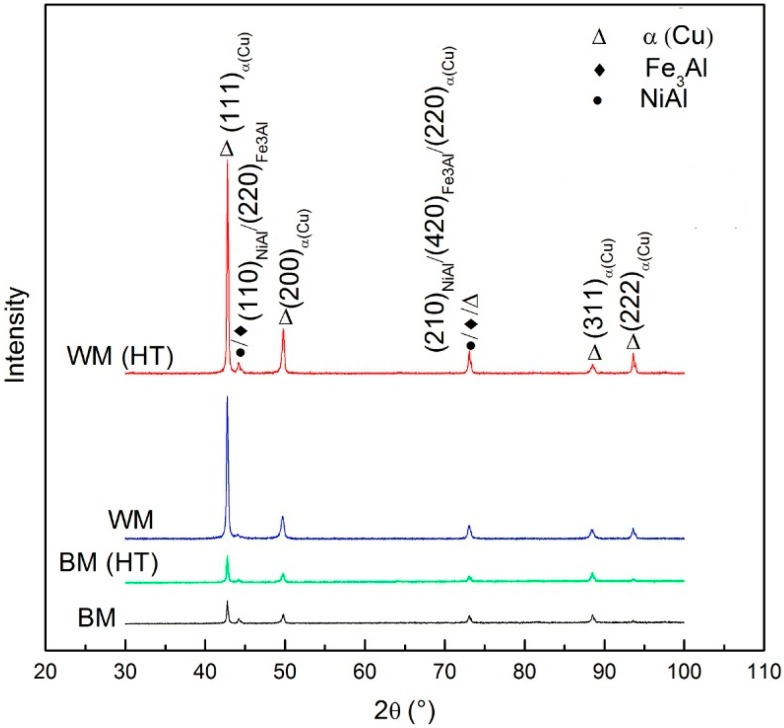
Comparison of phase constitutions of as-cast BM, as-deposited WM, heat-treated BM, and heat-treated WM.

In order to identify the phase structure, an XRD analysis was conducted in the cross-section of the components. A comparison of the XRD data from as-cast BM, heat treated BM, as-deposited WM, and heat treated WM is shown in Figure 6. All the diffraction patterns are found to contain a prominence of α(Cu) phase and a certain amount of NiAl or Fe_3_Al precipitates. In comparison to the BM, the volume fraction of precipitates decreases significantly in the WM due to the suppressed eutectoid reaction β → α + κ_III_ by the rapid cooling rate. With the PWHT, there is little change in BM, however, more intermetallic phases are precipitated in the WM, which is consistent with microstructure observations. 

**Figure 7:**
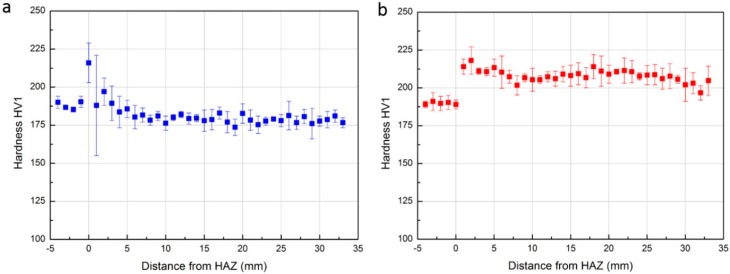
Microhardness profile in the cross-section as the function of the distance from HAZ. (**a**) Before PWHT; (**b**) After PWHT.

Microhardness testing was performed to understand the effect of fusion welding and PWHT on the hardness of the samples. The microhardness profile of the sample before PWHT, as the function of distance from the HAZ along the wall build direction, is shown in Figure 7a. The BM (distance from −5 to 0 mm) showed little variation in readings with the mean value of 188 HV1. This is expected due to the uniform microstructure of the BM. The HAZ (at 0 mm) in samples showed higher values of hardness due to the formation of martensite. Large variations were observed as there exist partially-transformed martensitic products and the width of HAZ is generally slightly larger than the diagonal of indention left by Vickers Hardness Test. It should be noted that the largest variations of hardness were shown in the region of the first deposited layer (at distance approximately 1 mm) as the microstructure in this region underwent significant change due to the different heat input and cooling rate. The remainder region of as-deposited WM (distance >2 mm) shows very uniform hardness (averaged to 181 HV1) which is also comparable to the BM. With PWHT, as shown in Figure 7b, the hardness in BM shows little change, while in the HAZ, shifts to the same value as the BM. In WM, the hardness is improved around 15% from 181 to 210 HV1 which is favorable. This is due to the refined grains (as shown in Figure 5g) and more precipitations of intermetallic in α phase (as shown in Figure 6). 

**Figure 8:**
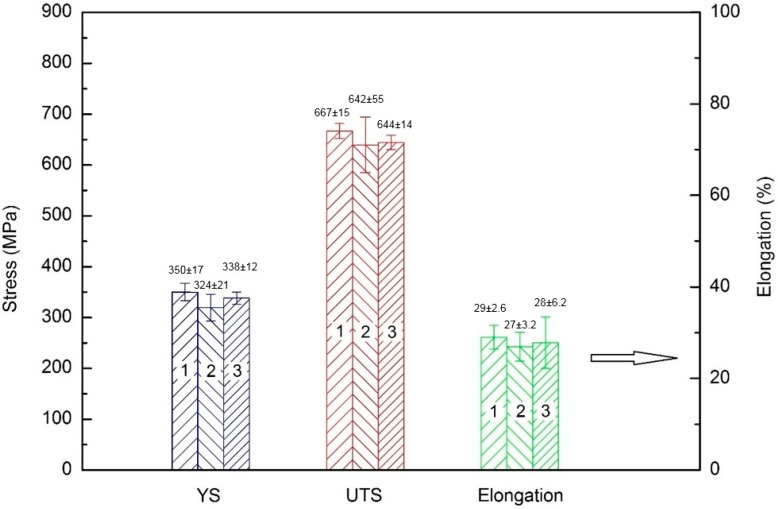
Effects of heat input on mechanical properties of as-deposited WM. Each data point represents the average of more than 10 specimens from one of three welding heat inputs.

The room temperature tensile properties of the as-deposited NAB are shown in Figure 8. Samples from three deposited walls, using different welding parameters, are named “1”, “2”, and “3”, respectively as shown in Figure 8. A uniform yield strength (YS), ultimate tensile strength (UTS), and elongation (%) are found in all three testing walls with the variations in average values being approximately 26 MPa, 35 MPa, and 2%, respectively. This indicates that the effect of heat input on the mechanical properties of the additively manufactured component is insignificant. This is desirable as a wide range of welding parameters can be used for the WAAM of NAB while maintaining uniform mechanical properties. 

**Figure 9:**
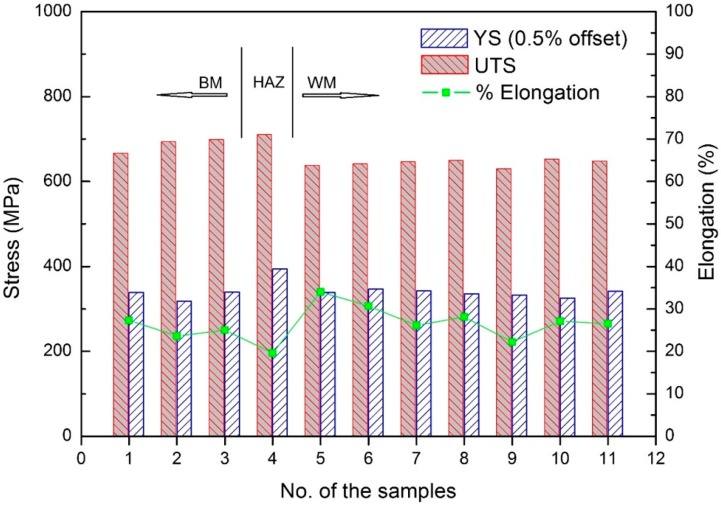
Location effect on mechanical properties of the NAB component produced by WAAM.

As the walls were deposited layer-upon-layer, it is interesting to understand the variation in mechanical properties along the wall build direction. Figure 9 shows the mechanical properties of samples taken from one of the as-deposited walls at different vertical locations. As expected, the BM has uniform mechanical properties with the average values of YS, UTS, and elongation being 332 MPa, 689 MPa, and 25.3%, respectively. In the HAZ, the higher YS of 394 MPa and higher UTS of 711 MPa, while a lower elongation of 19.6% was found, which is predictable. The values of UTS and YS in the WM span 339–342 MPa and 630–653 MPa, respectively. There is no significant variation (less than 3.6%) in tensile strength and yield strength in WM which is plausible. The average elongation in WM is 27.8%. The microstructure in the HAZ is predominantly partially transformed eutectoid structure, in particular, in the area close to the fusion line. The heat input is high enough to raise the temperature above the eutectic point, and therefore forming the β phase. This was retained with rapid cooling, resulting in a martensitic structure that is harder than the α phase, but also less ductile and more brittle. The mechanical properties across the weld metal are expected to be reasonably consistent. From the above data analysis, it is revealed that the mechanical properties of as-deposited WM are comparable to those of as-cast BM. 

**Figure 10:**
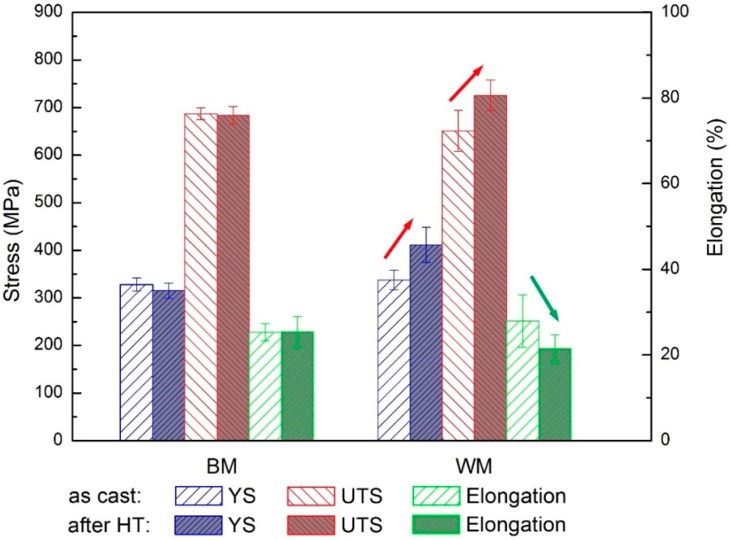
Effects of heat treatment on mechanical properties of as-cast BM and as-deposited WM. Each data point represents the average of samples from three different welding parameters, corresponding to the three-wall samples that were produced.

To investigate the effects of PWHT on mechanical properties of both as-cast and as-deposited NAB, a comparison of each tensile experiment was performed with the results shown in Figure 10. The data from different heat inputs were collected together with each data point in Figure 10 representing the average value of samples from three different welding parameters. Figure 10 shows that: (1) mechanical properties of as-deposited WM are comparable to those of as-cast BM; and (2) with the PWHT, there is little change in cast NAB components, while there is an improvement in tensile strength and a decrease in elongation of PWHT WM. The improved mechanical properties of PWHT WM are mainly due to the refinement in grain size and precipitations in the α phase during the heat treatment. These were confirmed by XRD results and microstructural observations. Therefore, the WAAM technique is able to produce NAB components with comparable or even superior mechanical properties than casting technology. It should be noted that the cast plate has the thickness of 30 mm that would not induce severe casting porosity. For marine applications, potential porosities trapped in large components would reduce mechanical properties, while that would not be an issue in wire arc additively-manufactured components.

**Figure 11:**
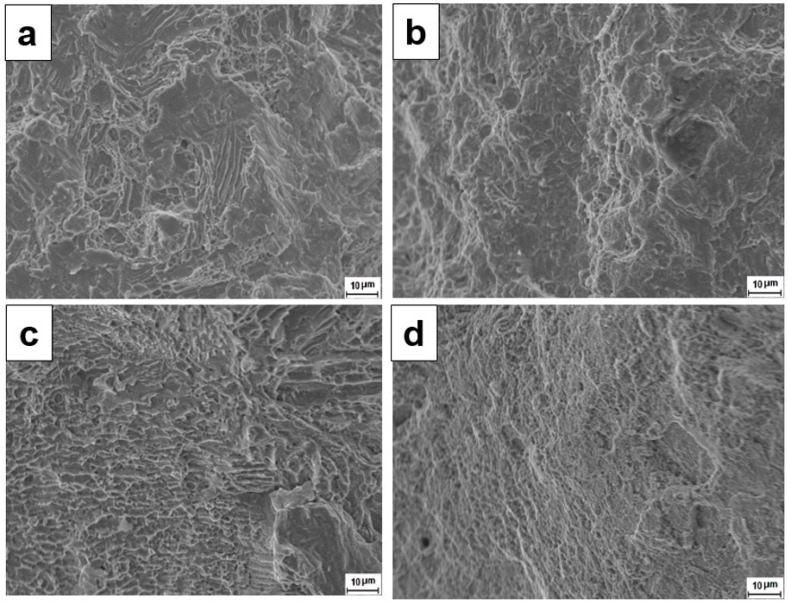
SEM fractographs of (**a**) as-cast BM; (**b**) heat treated BM; (**c**) as-deposited WM; and (**d**) heat treated WM.

Representative fracture surface of the tensile samples are shown in Figure 11a–d. The fracture morphologies in as-cast BM and heat treated BM after tensile tests are quite similar. In all cases, the fracture surfaces exhibit a mixture of brittle cleavage model and microvoid coalescence with all elongations greater than 20%. The as-deposited WM contains predominantly Widmanstätten α. The fracture surface exhibits the feature of lath structure, indicating the fracture preferentially propagates along the lath boundaries. The heat treatment relieves residual stresses and homogenizes the microstructure in the weld metal. Figure 5 clearly shows that the heat treatment decomposed the basket weave-like Widmanstätten structure, leaving a fine, homogenized microstructure of α phase and κ precipitates. The fine dimples on the fracture surface are consistent with the microstructure. 

## 4. Conclusions

The intent of this work is to demonstrate that the WAAM technique has the potential to fabricate functional NAB alloy. We have shown that fully dense NAB components were successfully produced using WAAM with various welding parameters. The variation of mechanical properties among all weld metals was insignificant. The as-deposited materials exhibits relatively fine microstructures, as well as comparable mechanical properties, with respect to as-cast NAB alloy. PWHT further refined the microstructure, improved tensile strength and hardness in the weld metal, revealing that WAAM is a promising alternative for manufacturing superior NAB components. Extending this work would involve investigating corrosion behavior of the additively-manufactured NAB alloy and the fabrication of near-net-shaped NAB components for marine applications.

## Figures and Tables

**Table 1:** Composition of NAB-based metal and welding wire (wt %).

Element	Cu	Al	Ni	Fe	Mn	Si	Zn	Sn	Pb	Mg	Cr
Base Metal	Rem	8.8	5.2	4.4	1.1	0.07	0.02	0.02	<0.01	<0.01	<0.01
Welding Wire	Rem	8.5–9.5	4–5.5	3–5	0.6–3.5	0.1	0.1	0.1	0.02		

**Table 2:** Welding parameters of three tests.

Test No.	Wire Feed Rate (m/min)	Travel Speed (mm/min)	Average Current (A)	Average Voltage (V)	Heat Input (J/mm)
1	5.4	400	175.5	24.8	653
2	6.7	400	218.3	26.7	874
3	8	400	256.1	29.0	1114

## References

[1] Brezina P. (1982). Heat treatment of complex aluminium bronzes. Int. Mater. Rev..

[2] Culpan E., Rose G. (1978). Microstructural characterization of cast nickel aluminium bronze. J. Mater. Sci..

[3] Yu H., Zheng Y., Yao Z. (2009). Cavitation erosion corrosion behaviour of manganese-nickel-aluminum bronze in comparison with manganese-brass. J. Mater. Sci. Technol..

[4] Ferrara R., Caton T. (1982). Review of dealloying of cast aluminum bronze and nickel-aluminum bronze alloys in sea water service. Mater. Performance.

[5] Fuller M., Swaminathan A., Zhilyaev A., McNelley T. (2007). Microstructural transformations and mechanical properties of cast NiAl bronze: Effects of fusion welding and friction stir processing. Mater. Sci. Eng. A.

[6] Wu Z., Cheng Y.F., Liu L., Lv W., Hu W. (2015). Effect of heat treatment on microstructure evolution and erosion–corrosion behavior of a nickel–aluminum bronze alloy in chloride solution. Corros. Sci..

[7] Ma Z. (2008). Friction stir processing technology: A review. Metall. Mater. Trans. A.

[8] Li H., Grudgings D., Larkin N., Norrish J., Callagan M., Kuzmikova L. (2012). Optimization of welding parameters for repairing NiAl bronze components. Mater. Sci. Forum.

[9] Almeida P.S., Williams S. Innovative process model of Ti-6Al-4V additive layer manufacturing using cold metal transfer (CMT). Proceedings of the Twenty-First Annual International Solid Freeform Fabrication Symposium.

[10] Mok S.H., Bi G., Folkes J., Pashby I., Segal J. (2008). Deposition of Ti–6Al–4V using a high power diode laser and wire, part II: Investigation on the mechanical properties. Surf. Coat. Technol..

[11] Brandl E., Palm F., Michailov V., Viehweger B., Leyens C. (2011). Mechanical properties of additive manufacturing titanium (Ti-6Al-4V) blocks deposited by a solid-state laser and wire. Mater. Des..

[12] Ding D., Pan Z., Cuiuri D., Li H. (2015). Wire-feed additive manufacturing of metal components: Technologies, developments and future interests. Int. J. Adv. Manuf. Tech..

[13] Wang F., Williams S., Rush M. (2011). Morphology investigation on direct current pulsed gas tungsten arc welded additive layer manufactured Ti6Al4V alloy. Int. J. Adv. Manuf. Tech..

[14] Shen C., Pan Z., Cuiuri D., Roberts J., Li H. (2016). Fabrication of Fe-FeAl Functionally Graded Material Using the Wire-Arc Additive Manufacturing Process. Metall. Mater. Trans. B.

[15] Sabbaghzadeh B., Parvizi R., Davoodi A., Moayed M.H. (2014). Corrosion evaluation of multi-pass welded nickel–aluminum bronze alloy in 3.5% sodium chloride solution: A restorative application of gas tungsten arc welding process. Mater. Des..

[16] Wharton J.A., Barik R.C., Kear G., Wood R.J.K., Stokes K.R., Walsh F.C. (2005). The corrosion of nickel–aluminium bronze in seawater. Corros. Sci..

[17] Liu C.M., Tian X.J., Tang H.B., Wang H.M. (2013). Microstructural characterization of laser melting deposited Ti–5Al-5Mo–5V–1Cr–1Fe near β titanium alloy. J. Alloys Compd..

[18] Hasan F., Jahanafrooz A., Lorimer G.W., Ridley N. (1982). Morphology, crystallography, and chemistry of phases in as-cast nickel-aluminium bronze. Metal. Trans. A.

